# JAK inhibitors to treat STAT3 gain-of-function: a single-center report and literature review

**DOI:** 10.3389/fimmu.2024.1400348

**Published:** 2024-08-23

**Authors:** Faranaz Atschekzei, Stephan Traidl, Julia Carlens, Katharina Schütz, Sandra von Hardenberg, Abdulwahab Elsayed, Diana Ernst, Linus Risser, Thea Thiele, Theresa Graalmann, Juliana Raab, Ulrich Baumann, Torsten Witte, Georgios Sogkas

**Affiliations:** ^1^ Department of Rheumatology and Immunology, Hannover Medical School, Hannover, Germany; ^2^ Cluster of Excellence RESIST (EXC 2155), Hannover Medical School, Hannover, Germany; ^3^ Department of Dermatology and Allergy, Hannover Medical School, Hannover, Germany; ^4^ Department of Pediatric Pneumology, Allergy and Neonatology, Hannover Medical School, Hannover, Germany; ^5^ Department of Human Genetics, Hannover Medical School, Hannover, Germany; ^6^ Junior Research Group for Translational Immunology, TWINCORE, Centre for Experimental and Clinical Infection Research, a joint venture between the Helmholtz Centre for Infection Research and the Hannover Medical School, Hannover, Germany; ^7^ Biomedical Research in Endstage and Obstructive Lung Disease Hannover (BREATH), Member of the German Centre for Lung Research (DZL), Hannover, Germany; ^8^ Institute for Diagnostic and Interventional Radiology, Hannover Medical University, Hannover, Germany

**Keywords:** STAT3 gain-of-function, JAK inhibitors, baricitinib, ruxolitinib, tofacitinib, arthritis, antiphospholipid syndrome, trachyonychia

## Abstract

**Objective:**

The signal transducer and activator of transcription 3 (STAT3) gain-of-function (GOF) syndrome (STAT3-GOF) is an inborn error of immunity (IEI) characterized by diverse manifestations of immune dysregulation that necessitate systemic immunomodulatory treatment. The blockade of the interleukin-6 receptor and/or the inhibition of the Janus kinases has been commonly employed to treat diverse STAT3-GOF-associated manifestations. However, evidence on long-term treatment outcome, especially in the case of adult patients, is scarce.

**Methods:**

Clinical data, including laboratory findings and medical imaging, were collected from all seven patients, diagnosed with STAT3-GOF, who have been treated at the Hannover University School, focusing on those who received a Janus kinase (JAK) inhibitor (JAKi). Previously published cases of STAT3-GOF patients who received a JAKi were evaluated, focusing on reported treatment efficacy with respect to diverse STAT3-GOF-associated manifestations of immune dysregulation and safety.

**Results:**

Five out of seven patients diagnosed with STAT3-GOF were treated with a JAKi, each for a different indication. Including these patients, outcomes of JAKi treatment have been reported for a total of 41 patients. Treatment with a JAKi led to improvement of diverse autoimmune, inflammatory, or lymphoproliferative manifestations of STAT3-GOF and a therapeutic benefit could be documented for all except two patients. Considering all reported manifestations of immune dysregulation in each patient, complete remission was achieved in 10/41 (24.4%) treated patients.

**Conclusions:**

JAKi treatment improved diverse manifestations of immune dysregulation in the majority of STAT3-GOF patients, representing a promising therapeutic approach. Long-term follow-up data are needed to evaluate possible risks of prolonged treatment with a JAKi.

## Introduction

The signal transducer and activator of transcription 3 (STAT3) gain-of-function (GOF) syndrome (STAT3-GOF) is an autosomal dominant disease characterized by complex immune dysregulation, including lymphoproliferation and autoimmunity (1, 2). The phenotypic variability of STAT3-GOF can be explained, at least in part, through variations in the GOF effect of *STAT3* mutations, which differentially affect the baseline and induced activity of STAT3 as well as its affinity to DNA (3). Immune dysregulation in STAT3-GOF has been associated with reduced peripheral counts or suppressive function of regulatory T cells (Treg) (4, 5). Recently, a mouse model of a human *STAT3* GOF mutation revealed dysregulated T-cell differentiation, biased towards Th1-skewing and IFNγ overproduction, which may represent an additional mechanism of immune dysregulation in STAT3-GOF (6).

According to the largest cohort study on the natural history of STAT3-GOF (*N* = 191), more than two-thirds of patients necessitated systemic treatment with immunomodulatory medications (2). Common manifestations of immune dysregulation leading to systemic immunomodulatory treatment include lymphoproliferative disease, enteropathy, autoimmune cytopenias, and interstitial lung disease (ILD). Precision therapy in STAT3-GOF includes the blockade of the interleukin-6 receptor (IL-6R) with tocilizumab (TCZ) and the inhibition of the Janus kinases (JAKs) (7). Among JAK inhibitors (JAKi), ruxolitinib and tofacitinib have been commonly employed, especially in case of pediatric patients (7, 8).

Data on the management of adult patients with STAT3-GOF are scarce. Available case studies suggest the effectiveness of nonselective JAK inhibitors in treating STAT3-GOF-associated immune dysregulation (7, 8). However, most reported patients are pediatric cases and the provided follow-up period is short. Long-term follow-up data, especially from treated adult patients, would be necessary in order to evaluate the effectiveness and safety of JAKi in STAT3-GOF and associated sequelae. Here, we aimed at summarizing our experience with JAKi in STAT3-GOF. Our report provides follow-up data from five patients with diverse forms of immune dysregulation that were treated with baricitinib and/or ruxolitinib. In addition, we provide a systematic review of the effectiveness and outcome of JAKi treatment in all reported patients thus far.

## Materials and methods

### Study cohort

For this single-center retrospective study, all genetically tested primary immunodeficiency (PID) patients attending the Immunology outpatient clinics of the Hannover Medical School were screened for the diagnosis of STAT3-GOF. Genetic testing was performed by means of targeted next-generation sequencing (tNGS) of a panel of genes including *STAT3*, whole exome sequencing (WES) and/or whole genome sequencing (WGS), as reported previously (9–11). All clinical data, including laboratory findings and medical imaging, were collected from January 2017 to December 2023 from patients’ medical files. Clinical diagnosis of PID was based on the current European Society for Immunodeficiencies (ESID) diagnostic criteria (available at http://esid.org/Working-Parties/Registry/Diagnosis-criteria). PID-associated phenotypes were documented as described previously (12). In particular, those included autoimmune cytopenias, such as autoimmune hemolytic anemia (AIHA), idiopathic thrombocytopenic purpura (ITP), and organ-specific autoimmunity (insulin-dependent diabetes mellitus, thyroidopathies, atrophic gastritis, and arthritis). ILD was diagnosed based on typical CT scan findings, in the absence of evidence for an infectious or alternative cause. Splenomegaly was defined as spleen enlargement on ultrasound (≥11 cm). Lymphadenopathy was detected on palpation, ultrasound, CT, or magnetic resonance scan. Enteropathy included all cases of biopsy-proven non-infectious inflammatory bowel disease (IBD) (ulcerative colitis and Crohn’s disease), celiac disease, lymphocytic infiltration of the interepithelial mucous, the lamina propria, and/or the submucosa. All previous immunomodulatory treatments were documented from patients’ medical files. Therapeutic interventions such as TCZ or JAKi were given as part of routine clinical care of patients. Response to treatment was treatment was classified into complete, partial, and no response based on review of available medical files from at least two authors. The study was conducted in accordance with the recommendations of the Declaration of Helsinki and was approved by the Ethics Committee of the Hannover Medical School (approval number 5582, 11223**-**BO**-**K**-**2024).

### Systematic review of published cases treated with a JAK inhibitor

A systematic electronic search was performed in February 2024 in PubMed/MEDLINE and Google Scholar databases, using the search term “STAT3 gain of function” or “inborn errors of immunity and Janus kinase inhibitor”, to identify published studies reporting outcomes related to JAKi treatment for STAT3-GOF. The inclusion criteria were as follows: (a) reports on patients with STAT3-GOF under treatment with a JAKi and (b) reports providing statement on patient outcome under JAKi. The report by Leiding et al. (2) was excluded from analysis, as efficacy of JAKi in individual patients could not be extracted. An independent review of each paper articles was done by at least two authors (GS, FA, JC, and ST). For each case, the following data were extracted: *STAT3* mutation, sex, age at reporting, JAKi and concomitant immunomodulatory medications, follow-up during treatment with a JAKi, reported manifestations of STAT3-GOF, stated treatment effectiveness with respect to all reported manifestations, reported adverse events during treatment, and patient status at reporting [dead, alive, and hematopoietic stem cell transplantation (HSCT)]. Effectiveness of JAKi-based treatment with respect to STAT3-GOF-associated manifestations was classified into complete, partial, and no response, based on available clinical data and statements on patient outcome in original manuscripts, which were documented and judged by at least two authors. Disease was classified as JAKi refractory in the presence of at least one manifestation not responding to JAKi treatment or in case treated patients underwent HSCT, despite previous treatment with a JAKi. For deceased patients, documented causes of death were extracted.

### Statistical analysis

For statistical calculation, we used GraphPad Prism 9 (GraphPad, La Jolla, USA). Descriptive statistics are reported as median and interquartile range (IQR) in case of continuous variables and as counts and percentages for dichotomous variables. Categorical variables were compared by the Yate’s continuity corrected chi-square test.

## Results

### *STAT3* variants and clinical manifestations

**Figure 1:**
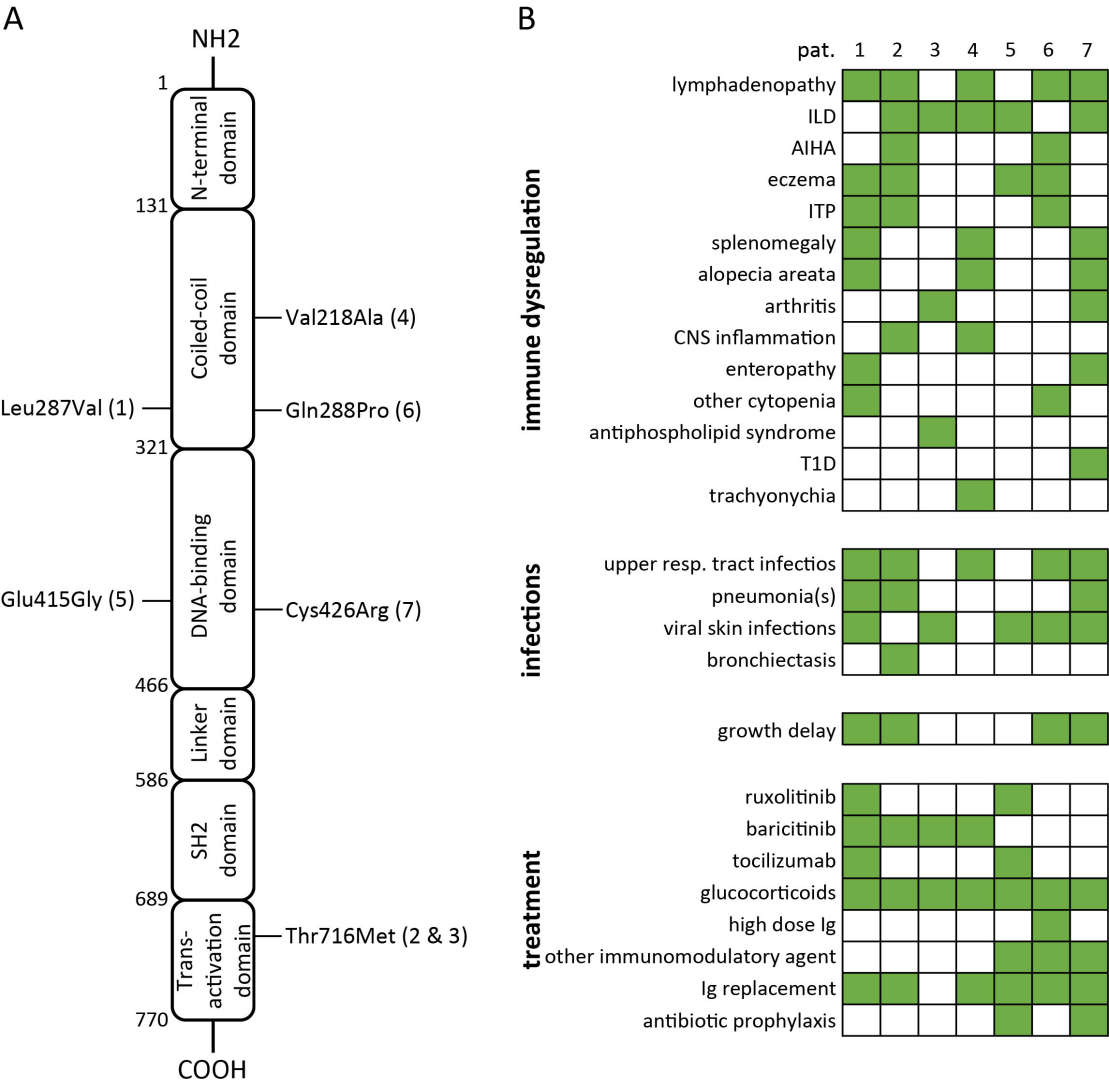
Summary of variants in *STAT3*, causing STAT3-GOF **(A)** and associated clinical manifestations as well as treatments **(B)** in seven patients (AIHA, autoimmune hemolytic anemia; CNS, central nervous system; Ig, immunoglobulin; ILD, interstitial lung disease; ITP, immune thrombocytopenia; T1D, type 1 diabetes).

Out of 411 patients with a previously diagnosed PID who have been subjected to genetic testing by means of diverse NGS methods (216 patients were exclusively tested with tNGS, 16 exclusively with WES, 101 exclusively with WGS, 44 with tNGS followed by WES, and 34 with tNGS followed by WGS), 4 unrelated patients had been diagnosed with STAT3-GOF (1%). An additional patient had been identified in the frame of a previous research project, employing tNGS to identify the genetic causes of secondary hypogammaglobulinemia in patients with rheumatic disorders (13). An infant was subjected to genetic testing due to treatment refractory congenital ILD. Finally, a boy with systemic immune dysregulation, including a treatment refractory ILD, was evaluated post mortem for an underlying primary immune regulatory disorder (PIRD). Identified *STAT3* mutations are presented in **Figure 1A**. Among a total of seven identified patients with STAT3-GOF, six were reported previously (see **Supplementary Table 1**).

Four out of seven patients with STAT3-GOF had been diagnosed with a PID, which could be classified as common variable immunodeficiency (CVID) (three out of seven patients) or unclassified antibody deficiency (one out of seven patients, i.e., patient 5). Three out of seven studied patients initially displayed no definite manifestation of immunodeficiency and developed hypogammaglobulinemia after the introduction of immunosuppressive treatment, including glucocorticoids and conventional synthetic disease-modifying antirheumatic drugs (csDMARDs). With the exception of a single patient (patient 4), presenting a late-onset rheumatoid arthritis (RA) at the age of 66 years as his first disease manifestation, all other patients (six of seven) displayed first STAT3-GOF-associated manifestation in childhood, more commonly at toddler age (three out of seven patients). The clinical manifestations of studied patients included ILD (five out of seven patients), lymphadenopathy and/or splenomegaly (four out of seven), autoimmune cytopenias (three out of seven), and arthritis (two out of seven). A female patient (patient 2) was diagnosed with antiphospholipid syndrome (APS) at the age of 39 years. An additional patient (patient 4) developed trachyonychia at the age of 28 years. Clinical manifestations of studied patients with STAT3-GOF are summarized in **Figure 1B**. A chronological summary of each case, including immunomodulatory medications employed to treat immune dysregulation, is shown in **Supplementary Figure 1**.

### STAT3-GOF-associated immune dysregulation leading to treatment with JAK inhibitors

Except for two patients, i.e., patient 6 and patient 7, a targeted treatment was employed in all other cases and included baricitinib (four out of five), ruxolitinib (two out of five), and TCZ (two out of five). TCZ treatment was started prior to JAKi in patient 1 and concurrently in patient 5. For patient 6, no targeted treatment has been employed so far, as sirolimus was started a year prior to the diagnosis of STAT3-GOF and led to satisfactory control of autoimmune pancytopenia and lymphadenopathy. In case of patient 7, diagnosis of STAT3-GOF was made only post mortem, which impeded consideration of treatment with TCZ or a JAKi. This patient displayed a progressive ILD, which was treated with high-dose pulse glucocorticoids, hydroxychloroquine, etanercept, and mycophenolate mofetil. All aforementioned drugs failed to control the rapid progression of ILD, which eventually led to lung transplantation. This patient died of respiratory failure due to a treatment refractory antibody-mediated transplant rejection at the age of 14.8 years, i.e., a year after lung transplantation.

**Figure 2:**
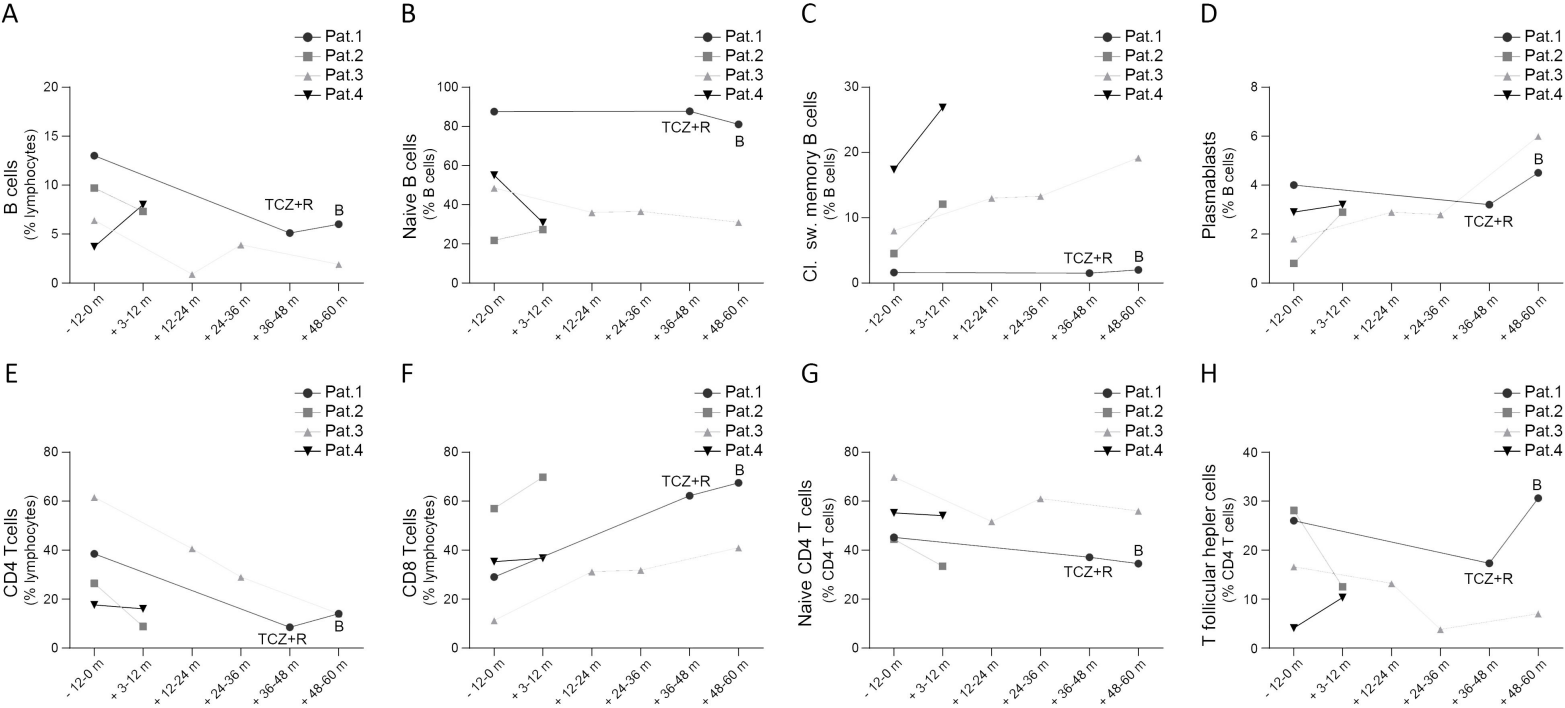
Peripheral B cell **(A-D)** and T cell subsets **(E-H)** in patients with STAT3-GOF prior and after introduction of JAKi treatment (B, baricitinib; m, month; pat, patient; TCZ, tocilizumab)

**Figure 3:**
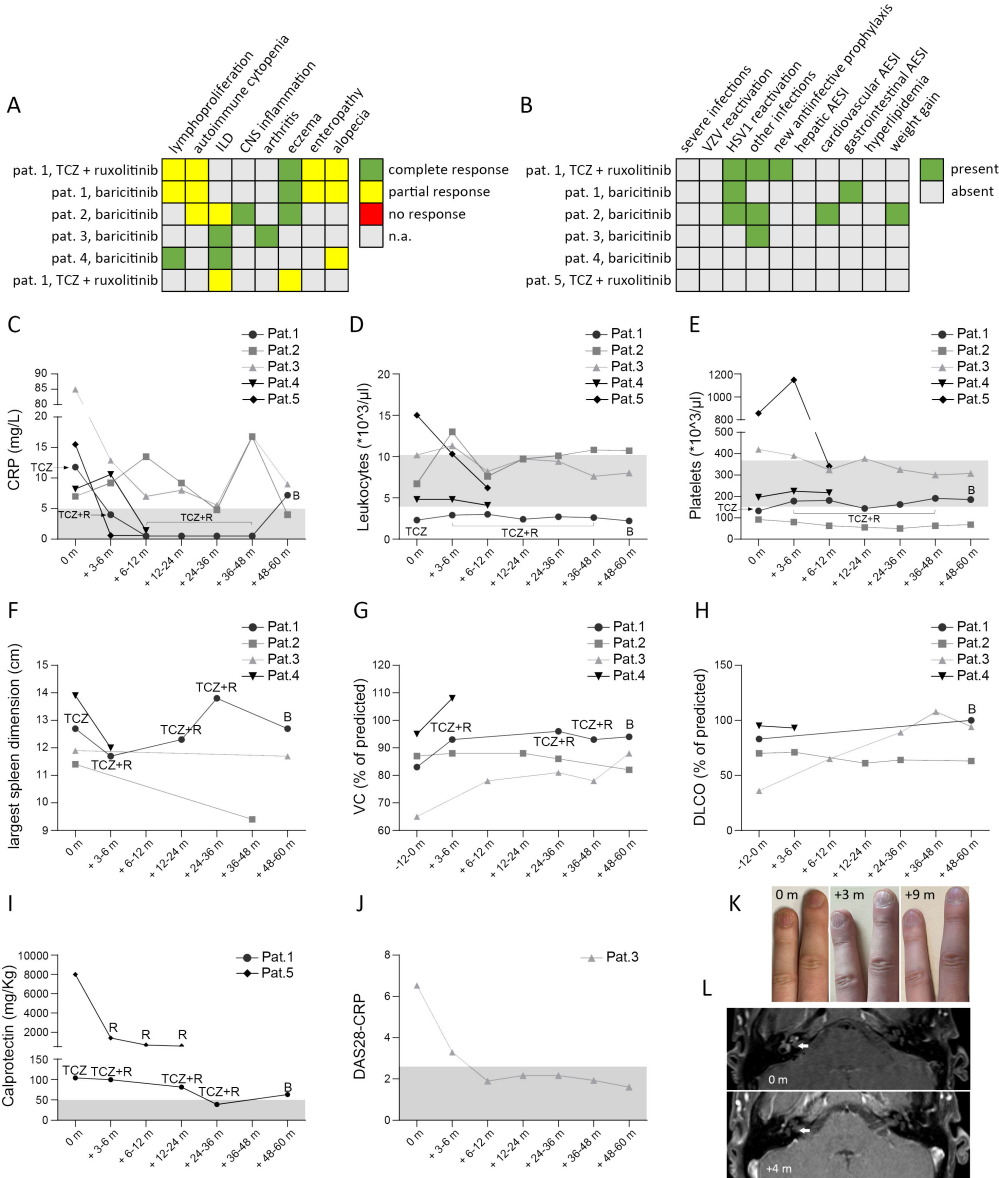
Outcome of treatment with a JAKi in 5 patients with STAT3-GOF. Summary of treatment response **(A)** and adverse events of special interest (AESI) **(B)**. Course of laboratory parameters, including C-reactive protein (CRP) **(C)**, leukocyte **(D)** and platelet counts **(E)**. For patient (pat.) 1 we indicate treatment, i.e. tocilizumab (TCZ), TCZ + ruxolitinib (R) and baricitinib (B), under which above mentioned laboratory values were measured. For patient 5 data from 1st year of treatment with ruxolitinib and TCZ are shown, prior to treatment suspension for lung transplantation. Sonographic evaluation of largest spleen dimension **(F)**. Course of lung function during treatment, including measurements of vital capacity (VC) and diffusion capacity for carbon monoxide (DLCO) (**G & H**, respectively). Measurement of calprotectin in stool **(I)**. For pat. 5 calprotectin was measured at diagnosis of chronic diarrhea, after suspending ruxolitinib treatment for suspected toxicity Evaluation of disease activity score 28 – CRP (DAS28-CRP) **(J)**. Trachyonychia and its course at 3 months (m) and 6 m after introduction of baricitinib **(K)**. Brain magnetic resonance imaging (MRI) showing cochlear inflammation (indicated by the white arrow) prior to starting baricitinib and 4 months (+4 m) after baricitinib introduction **(L)**.

Patient 1 was diagnosed with a collagenous colitis at the age of 1.4 years (**Supplementary Figure 1**). Later on, at the age of 9 years, she was diagnosed with an autoimmune enteropathy, which did not respond adequately to prednisolone pulse treatment. Recurrent episodes of immune thrombocytopenic purpura and autoimmune leukocytopenia were treated with repeated prednisolone pulses, which each time led to full recovery of platelet count, while recovery of leukocytes (lymphocyte and neutrophil counts) remained partial. Later, she developed chronic lymphadenopathy and splenomegaly. At the age of approximately 13 years, tNGS led to the diagnosis of STAT3-GOF. This genetic diagnosis, together with inadequate control of atopic eczema, autoimmune enteropathy, and autoimmune leukocytopenia, led initially to the introduction of treatment with TCZ in the form of monthly intravenous infusions (**Figures 3A, B**). Partial alleviation of gastrointestinal symptoms, eczema, and weight gain suggested a partial response to TCZ. However, persistence of alopecia, lymphadenopathy, splenomegaly, and leukocytopenia led to the addition of ruxolitinib nearly 1 year after the introduction of TCZ. Dual treatment with TCZ and ruxolitinib led initially to significant improvement of eczema, alopecia, and lymphadenopathy as well as splenomegaly, whereas leukocyte count did not improve and remained stable at approximately 2,500 cells/µL (**Figure 2D**). Approximately 4 years after the initiation of ruxolitinib and TCZ treatment, the patient again developed alopecia and lymphadenopathy. In addition, splenomegaly worsened (**Figure 2F**). Despite intensified immunoglobulin replacement resulting in an IgG trough serum level of 16 g/L, this patient developed recurrent upper respiratory tract infections. Because of the inadequate control of alopecia and autoimmune leukocytopenia, ruxolitinib treatment was switched to baricitinib. Furthermore, considering the fact that leukocytopenia and reported recurrent infections could represent adverse effects of the IL-6R blockade, TCZ was gradually withdrawn after prolongation of spacing between individual doses. Six months after treatment with baricitinib as a monotherapy, leukocyte count remained stably low, while alopecia, lymphadenopathy, and splenomegaly improved.

Patient 2 was diagnosed with CVID at the age of 24 years (**Supplementary Figure 1**). Shortly after, she had an episode of AIHA and later an episode of ITP, both of which responded to pulse glucocorticoid treatment. At the age of 33 years, she was diagnosed with an ILD, for which she received no treatment as she displayed no respiratory symptoms and pulmonary function parameters were stable. One year later, while the diagnosis of STAT3-GOF had been established, she suffered an acute sensorineural hearing loss accompanied by tinnitus and headache that recurred after empirical treatment with intravenous methylprednisolone pulse, initiated by her otolaryngologist. Diagnostic workup included cerebrospinal fluid analysis, which yielded no evidence for an infectious cause of her neurological symptoms. Brain magnetic resonance imaging (MRI) revealed an inflammatory cochlear lesion accompanied by weak disseminated white matter lesions in the fluid-attenuated inversion recovery (FLAIR) sequence. Considering the refractory course of her symptoms, the MRI findings, and the diagnosis of STAT3-GOF, we introduced baricitinib, which led to gradual improvement of neurological symptoms (**Figure 3A, B**). At follow-up, approximately 4 months after the introduction of baricitinib, glucocorticoids were withdrawn, while neurological symptoms and central nervous system (CNS) lesions disappeared (**Figure 3L**). While baricitinib was well tolerated, approximately 5 years after treatment introduction, this patient developed a stroke at the area supplied by the middle cerebral artery. At that point, MRI and computed tomography (CT) angiography revealed no evidence of CNS inflammation or vasculitis. Cardiac assessment, including a transesophageal echocardiography, revealed no pathological findings. Given the initially unclear etiology of stroke and the thromboembolic risk associated with JAKi treatment (14), we decided to stop baricitinib. Laboratory evaluation for a hypercoagulable state revealed the persistent detection of IgM anti-cardiolipin antibody, which was not derived from immunoglobulin replacement. In the absence of evidence for an alternative cause of stroke, we diagnosed an APS and started a long-term indefinite warfarin treatment.

Patient 3 had an unremarkable medical record until the age of 66 years (**Supplementary Figure 1**). At that age, he developed a chronic symmetrical polyarthritis affecting the MCP and PIP, diagnosed as a seronegative RA, which led to the introduction of treatment with prednisolone and methotrexate (MTX). Approximately 6 months after the introduction of MTX, this patient developed respiratory symptoms that led to withdrawal of MTX, due to a suspected MTX-induced lung toxicity. Examination of sputum and bronchoalveolar lavage (BAL) detected no pathogen. A chest x-ray performed prior to the onset of respiratory symptoms revealed a right basal streaky opacity that was rather suggestive of a preexisting ILD. Worsening of the respiratory symptoms together with chest CT findings revealing progressive ILD as well as persistence of polyarthritis led to the introduction of treatment with baricitinib (**Figures 3A, B**). Baricitinib led to sustained remission of arthritis and gradual improvement of respiratory symptoms, while enabling tapering of prednisolone (**Figure 3J**).

Patient 4 displayed diverse manifestations of immune dysregulation during his childhood (**Supplementary Figure 1**), including alopecia areata and disseminated inflammatory lesions in the brain, especially affecting subcortical areas of the temporal and occipital lobe as well as the cerebellar peduncle and the corpus callosum, which led to complex focal seizures. All aforementioned inflammatory manifestations were treated with glucocorticoids, including intravenous pulses. Diagnosis of STAT3-GOF was made at the age of 23 years, while this patient displayed no inflammatory or autoimmune manifestations, which would necessitate immunomodulatory treatment. At the age of 28 years, he developed a severe trachyonychia with longitudinally nail ridging and worsening onychoschizia. The latter together with alopecia led to the introduction of treatment with baricitinib, which led to a moderate hair regrowth and significant improvement of nail lesions (**Figures 3A, B, K**).

Patient 5 is a 2.5-year-old boy. He was born by vaginal delivery at 34 weeks’ gestation to a gravida 1, para 32-year-old healthy mother after maternal induction of labor due to intrauterine growth restriction (IUGR). He was diagnosed with congenital hypothyroidism. In addition, directly at birth, he developed severe hypercapnic respiratory failure, leading to invasive mechanical ventilation. Diagnostic workup of respiratory failure included a chest CT, which revealed bilateral diffuse ground-glass opacities with subpleural fibrosis. Diverse antibiotics, including ampicillin, gentamycin, cefotaxime, vancomycin, meropenem, and cotrimoxazole, failed to improve this patient’s respiratory situation, and tests for infectious agents, including *Pneumocystis jirovecii* and CMV, remained negative. The above findings led to the diagnosis of an ILD and the introduction of glucocorticoid treatment, initially with dexamethasone. Dexamethasone enabled the switch to noninvasive mechanical ventilation. Severe congenital ILD led to genetic testing by means of WES, which revealed a variant in *STAT3* that had been previously reported to be pathogenic (15), establishing the diagnosis of STAT3-GOF. Ruxolitinib and TCZ were started concurrently at the age of 3 months, stabilizing the pulmonary situation of the patient, who, however, kept requiring high-flow oxygen therapy and was listed for lung transplantation. At the age of 12 months, this patient developed hypogammaglobulinemia, which led to immunoglobulin replacement. Three months later, persistence of ILD led to lung transplantation. During the perioperative period, both ruxolitinib and TCZ were paused. After lung transplantation, he was set on standard immunosuppressive treatment with mycophenolate mofetil, tacrolimus, and prednisolone. Shortly after lung transplantation, he developed elevated liver enzymes and increasing C-reactive protein (CRP) values that normalized after reintroduction of both ruxolitinib and TCZ. After 2 monthly courses, TCZ was suspended for fear of severe infections. At the age of 2 years, he developed worsening anemia, leading to suspension of ruxolitinib due to a suspected toxicity. A month later, he developed eczema and chronic diarrhea that was associated with increasing CRP values and elevated fecal calprotectin. Colonic biopsy revealed a slight lymphoid hyperplasia. These findings suggested active immune dysregulation in the frame of STAT3-GOF, despite the standard immunosuppressive regime for lung transplantation, and led to the reintroduction of ruxolitinib at a higher dose [1.3 mg/kg/day, instead of 0.8 mg/kg/day (previous dose)]. This led to regression of diarrhea, eczema, and anemia.

### Retrospective assessment of efficacy and safety of JAK inhibitors

In the present study, we provide a mean follow-up of 38.4 months (minimum follow-up: 10.2 months in case of patient 4, maximum follow-up: 60 months in case of patient 3) of treatment with a JAKi in five patients with diverse STAT3-GOF-associated manifestations. Evaluation of laboratory parameters prior to and after introduction of treatment reveals a drop in the serum levels of CRP 3 to 9 months after the introduction of treatment in the case of four out of five treated patients, though in two patients (patients 1 and 5), this could rather reflect the inhibition of the IL-6R, i.e., the effect of concomitant TCZ treatment (**Figure 3C**). Despite persistently reduced leukocyte counts in the case of patient 1 and reduced platelet counts in the case of patient 2, all five patients displayed relatively stable full blood count parameters during treatment that did not lead to additional immunomodulatory treatment (**Figures 3D, E**). Retrospective evaluation of patients’ medical records suggests a beneficial effect of JAKi in controlling lymphoproliferation. With the exception of patient 1, retrospective evaluation of abdominal ultrasound findings suggests a reduction in the largest spleen dimension during JAKi treatment (**Figure 3F**). All four patients with a documented ILD displayed stable or improved lung function parameters during treatment with a JAKi (**Figures 3G, H**). However, in the case of patient 5, diffuse ILD and persistent respiratory failure led to lung transplantation. Autoimmune enteropathy in patient 1 associated with a mild increase in fecal calprotectin (**Figure 3I**). Despite normal or near-normal levels of fecal calprotectin, this patient kept reporting intermittent gastrointestinal symptoms, including abdominal pain and diarrhea, which suggested an inadequate control of enteropathy. Patient 5 developed enteropathy after stopping ruxolitinib, due to suspected toxicity. Reintroduction of ruxolitinib led to regression of diarrhea, which associated with a substantial drop in fecal calprotectin levels. For both patients with concomitant TCZ (patients 1 and 5), discontinuation of TCZ did not lead to worsening or new manifestations, suggesting control of immune dysregulation primarily through their JAKi treatment. Retrospective evaluation of peripheral lymphocyte subsets revealed increased percentages of class-switched memory B cells accompanied by increased plasmablast counts in three out of four treated patients for whom data were available, after the introduction of treatment with a JAKi (**Figures 2A–D**). In the case of T cells, all four treated patients displayed a drop in CD4 T-cell percentages that was accompanied by a drop in naïve CD4 T cells and an elevation in percentages of CD8 T cells (**Figures 2E–H**).

### A systematic review of JAK inhibitor treatment in STAT3 gain-of-function

**Figure 4:**
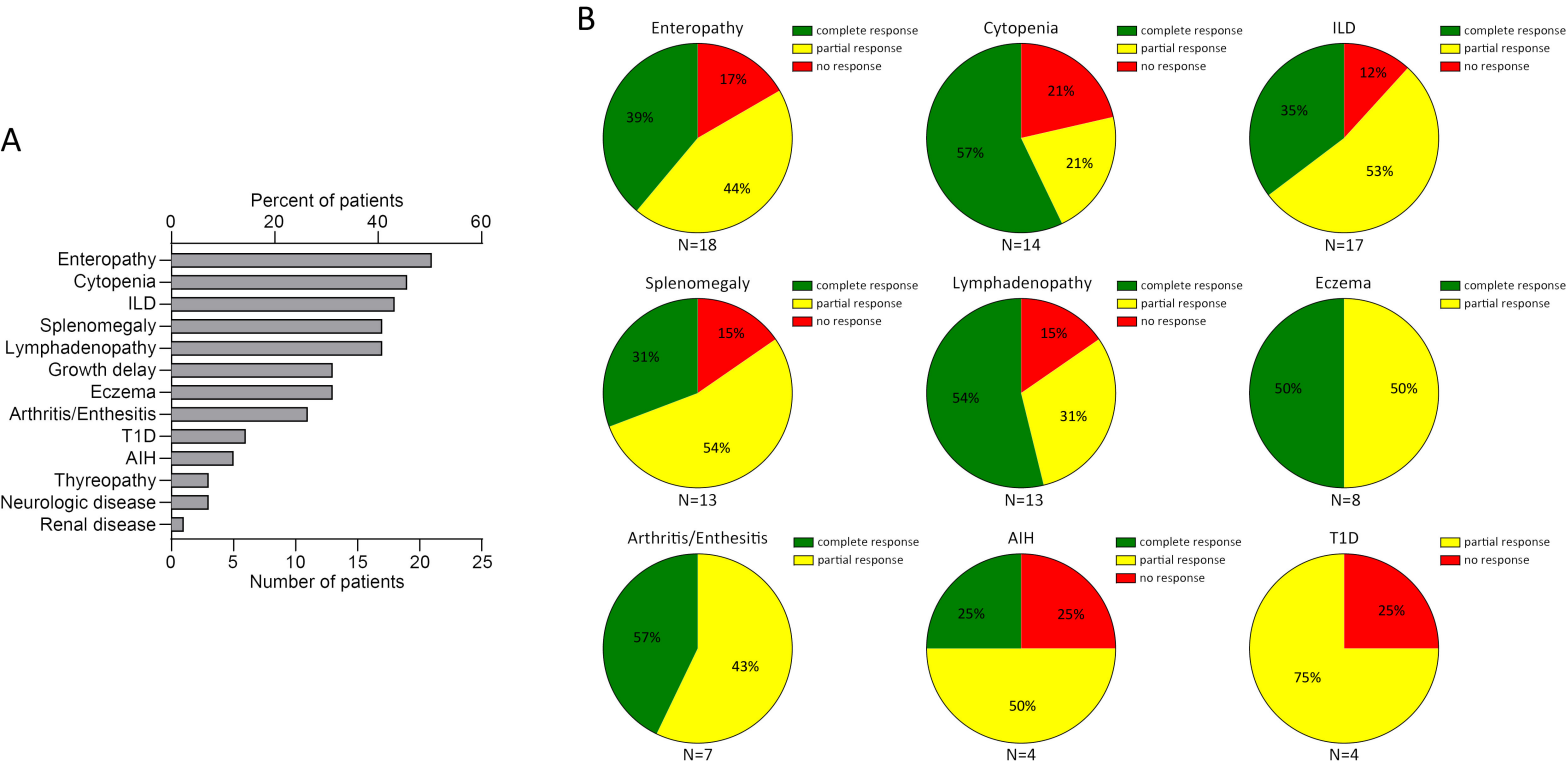
Summary of clinical manifestations **(A)** and review of response to a JAKi in 41 patients with STAT3-GOF **(B)** (AIH, autoimmune hepatitis; ILD, interstitial lung disease; n.a., not available; T1D, type 1 diabetes).

With the addition of the five cases presented here, the outcome of JAKi treatment has been reported for 41 patients with STAT3-GOF (**Supplementary Table 2**) (7, 8, 16–27). Duration of follow-up after introduction of a JAKi was provided for 34 out of 41 cases. Median follow-up duration was 18.5 months (IQR: 8, 32.5). STAT3-GOF-associated manifestations in evaluated patients are summarized in **Figure 4A**. Among those, the majority, i.e., 25 (61%), have been treated with ruxolitinib, 13 (31.7%) with tofacitinib, and 4 (9.8%, presented in this report) with baricitinib. A single patient (patient 1) was switched from ruxolitinib to baricitinib. The dose of JAKi was available for all but two patients (patients 1 and 34, **Supplementary Table 3**). The dose of baricitinib was based on the European Medicines Agency (EMA) recommendation for the treatment of patients with RA (28, 29). Daily dosage of tofacitinib ranged from 5 to 20 mg daily. With the exception of three patients (patients 11, 18, and 19), all received doses up to 10 mg daily. In the case of two patients (patients 19 and 27), dose was reduced due to reported adverse events. In the case of another patient (patient 13), however, dose was increased from 5 to 7.5 mg daily, which led to better disease control. For the majority of ruxolitinib-treated patients, reported daily dose was adjusted based on body surface area and varied substantially, ranging from 8 to 59 mg/m^2^ daily. Evaluation of the quotes of treatment-refractory cases between the subgroups of patients treated with up to 30 mg/m^2^ daily and those receiving higher doses suggests the lack of a clear dose–response relationship (5/14 vs. 3/9, odds ratio: 0.9, *p* = 0.9067). In the case of 2 patients, ruxolitinib dose was reduced due to reported adverse events (patients 19 and 27). A total of 9 out of 41 patients were also treated with TCZ, which was started before (5 patients), concurrent (3 patients), or after (1 patient) the introduction of a JAKi. In addition, in three patients, previous TCZ treatment failed to control disease and was stopped prior to the introduction of JAKi treatment (patients 18, 22, and 30), while in a single patient (patient 34), TCZ together with ciclosporin was reported to effectively control cytopenias that were refractory to ruxolitinib. There were no significant differences with respect to the quote of patients with treatment-refractory disease across the subgroups of patients receiving different JAKi (**Table 1**), which may suggest the lack of significant differences in the effectiveness of the three tried JAKi. Similarly, the localization of the mutation at the different domains of STAT3 does not seem to affect response to JAKi treatment. Common manifestations that were followed up during treatment with a JAKi included enteropathy (*N* = 18, 43.9%), cytopenias (*N* = 14, 31.1%), splenomegaly (*N* = 13, 31.7%), and ILD (*N* = 17, 41.5%) (**Figure 4B**). With the exception of autoimmune hepatitis and diabetes, either a partial or a complete treatment response has been reported for the majority of treated patients. A fatal outcome was reported in the case of four patients who received a JAKi. Death was reported to be related to previous HSCT in one patient (patient 9). For another patient, cause of death was reported as “unrelated to the STAT3-GOF symptoms” (patient 22). A 14-year-old female patient with life-threatening immune dysregulation and concomitant immunosuppressive treatment (sirolimus or mycophenolate) developed complications like sepsis and diffuse intravascular coagulation (DIC) and died a month after the introduction of JAKi (patient 7). The fourth patient with a fatal outcome was a 15-year-old male patient with severe ILD, who died after introduction of ruxolitinib due to respiratory failure complicated by pulmonary hemorrhage and infectious complications (aspergillosis, *Candida glabrata*, and *Pseudomonas* sepsis) (patient 35). Follow-up period during JAKi treatment was not determined for this case. Including a fatal outcome, adverse events were reported in the case of 21 out of 41 patients (51.2%, **Supplementary Table 3**). Infectious complications were reported in nine patients. Those included sepsis (patients 32 and 35) and thoracic herpes zoster (patient 23). For the rest of the patients, the reported infectious complications were rather mild. Regarding anti-infective prophylactic treatment, a total of 20 patients (48.8%) were under immunoglobulin replacement, 11 patients were receiving prophylactic cotrimoxazole treatment (26.8%), 4 were under prophylactic antiviral treatment (9.8%, drug not specified), and a single patient (2.4%) was receiving antimycotic treatment. Immunoglobulin replacement was introduced in all 20 patients prior to the introduction of the JAKi treatment. In 5 out of 11 treated patients, cotrimoxazole treatment was related to the introduction of JAKi treatment. The same was the case with the majority of patients receiving antiviral prophylaxis (i.e., three out of four). However, the exact time point, dose, and reason for the introduction of prophylactic antibiotics or antiviral was not reported. Unintentional weight gain was reported in five patients (patients 19, 30, 37, 39, and 40) and led to the reduction of the dose of ruxolitinib in one of them (patient 39). Adverse events, such as cytopenias in two patients (patients 14 and 34), psoriasis (patient 12), and APS-associated stroke (patient 40), could represent STAT3-GOF-related manifestations and rather reflect the incomplete efficacy of JAKi treatment.

**Table 1:** Efficacy of treatment, depending on employed JAKi and localization of mutation in *STAT3*.

Variable	No JAKi—refractory immune dysregulation (*N* = 31)*	At least one JAKi—refractory manifestation of immune dysregulation (*N* = 12)	OR (95% CI)	*p*
Tofacitinib (*N* = 13)	10	2	2.5 (0.44–12.89)	0.4826
Ruxolitinib (*N* = 25)	15	9	0.33 (0.09–1.49)	0.2568
Baricitinib (*N* = 4)	3	1	1.22 (0.17–17.17)	0.6777
NTD (*N* = 2)	2	0	n.a.	0.8918
CCD (*N* = 8)	5	3	0.62 (0.11–2.78)	0.8908
DBD (*N* = 18)	15	3	3.21 (0.71–12.45)	0.2213
SH2D (*N* = 4)	2	2	0.37 (0.05–2.68)	0.7033
TAD (*N* = 9)	5	4	0.42 (0.08–1.66)	0.4727

*For evaluation of response to different JAKi, number of cases was 32 (N = 32), as for patient 10, sequential treatment with ruxolitinib and baricitinib was evaluated separately.

CCD, coiled-coil domain; CI, confidence interval; DBD, DNA-binding domain; JAKi, Janus kinase inhibitor; n.a., not available; NTD, NH2-terminal domain; SH2D, Src-homology 2 domain; OR, odds ratio; TAD, transcription activation domain.

## Discussion

In the present report, we retrospectively evaluated the outcome of JAKi treatment in four adults and a child with STAT3-GOF. Our findings suggest that JAKi may be an effective treatment for diverse STAT3-GOF-associated manifestations. Noteworthy is the complete remission of CNS inflammation (patient 2), arthritis (patient 3), and ILD (patient 3) as well as the improvement of trachyonychia (patient 4) under baricitinib, whose effectiveness for STAT3-GOF-associated immune dysregulation has not been reported previously. However, with the exception of a single patient, all other treated patients continued to display manifestations of immune dysregulation, such as alopecia, splenomegaly, and/or cytopenias. In addition, in one patient, autoimmunity in the form of APS manifested for the first time during treatment with baricitinib. To our knowledge, the reported manifestations of trachyonychia and APS have not been previously associated with STAT3-GOF and may therefore expand its phenotypic spectrum. Considering all seven patients with STAT3-GOF, in the case of three of them, no PID was diagnosed previously and hypogammaglobulinemia developed only after the introduction of immunosuppressive medications, which would be consistent with a secondary hypogammaglobulinemia. This suggests that conventional immune modulation with glucocorticoids or csDMARDs may precipitate immunodeficiency in STAT3-GOF. On the other hand, primary antibody deficiencies belong to the phenotypic spectrum of STAT3-GOF. Consequently, STAT3-GOF exemplifies an IEI blurring the dichotomous classification of hypogammaglobulinemia into primary and secondary (13, 30). In addition to the broad phenotypic spectrum of STAT3-GOF, our report highlights the very variable disease onset, ranging from congenital disease to first presentation at the seventh decade of life, in the present series of cases. The latter together with the expected better outcomes of timely diagnosed children, who can profit from targeted treatments, makes STAT3-GOF an increasingly relevant diagnosis for physicians treating adult patients as well.

According to the largest presented cohort of STAT3-GOF patients thus far, which included all here-reported cases, 40 out of 191 patients were reported to be treated with a JAKi (2). However, data collection for this report did not include evaluation of efficacy of JAKi treatment or possible adverse events. Recently, Fischer at al. evaluated the efficacy and safety of JAKi treatment in a retrospective study, including 21 patients with STAT3-GOF, who were treated with either tofacitinib or ruxolitinib, the majority of whom (17/21) were pediatric patients (8). In this study 7 out of 21 (i.e., 33%) patients, all pediatric ones, achieved complete remission of STAT3-GOF-associated manifestations, whereas the rest displayed disease activity, more commonly in the form of lymphoproliferation or ILD, despite treatment with a JAKi. In addition, Forbes et al. and Kaneko et al. reported a clinical improvement with JAKi in six and four pediatric patients, respectively (7, 16). Evaluation of all available reports, providing follow-up data after treatment introduction, suggests that JAKi can lead to an improvement or reversal of severe immune dysregulation, including cytopenias, enteropathy, ILD, and arthritis (**Figure 4**). However, a complete remission of all STAT3-GOF-associated manifestations of immune dysregulation was reported for a minority of treated patients (i.e., 24.4%). The latter may be the consequence of a relatively delayed introduction, i.e., after the permanent tissue damage has occurred, for example, in the case of T1D or ILD. In line with these findings, the youngest patient in this cohort necessitated lung transplantation due to advanced ILD, despite very early diagnosis of STAT3-GOF at the age of 3 months and consequently early introduction of JAKi treatment. In addition, persistence of autoimmune or lymphoproliferative manifestations might reflect the residual steady-state or induced hyperactivation of STAT3, which cannot be completely reversed through the upstream inhibition of JAKs, suggesting the need for development of novel therapeutic approaches that would directly target STAT3.

Among JAKi, the dual JAK1 and JAK2 inhibitor ruxolitinib has been more commonly employed to treat STAT3-GOF. Systemic, particularly twice-daily oral ruxolitinib is both Food and Drug Administration (FDA)- and EMA-approved for hematological indications, i.e., primary myelofibrosis, polycythemia vera, and graft-versus-host (GvHD) disease (31–33). Similar to ruxolitinib, baricitinib, which has been more commonly employed in our center, is a dual JAK1 and JAK2 inhibitor. In contrast to ruxolitinib, approved indications for baricitinib (28, 29) fall under the clinical spectrum of STAT3-GOF, which may enable its in-label use. In particular, once-daily oral baricitinib is FDA- and EMA-approved for the treatment of RA. In addition, it is EMA-approved for the treatment of moderate-to-severe atopic dermatitis, active juvenile idiopathic arthritis, and alopecia areata.

Dosing of JAKi varied especially in the case of ruxolitinib. While tofacitinib and baricitinib dosing was commonly based on licensed indications, the exact dosing strategy for ruxolitinib, including the choice of starting dose, remains unclear. According to the report by Fischer et al., ruxolitinib was started in the majority of patients with the target dose, while in others, a ramp-up scheme was followed (8). Similar to tofacitinib, collected data suggest that disease severity and treatment tolerance may have defined the dose of ruxolitinib. Reported data did not allow a clear dose–treatment response evaluation. However, our systematic literature review suggests that very high doses of ruxolitinib or tofacitinib (higher than 30 mg/m^2^/day and 10 mg/day, respectively), may not increase treatment effectivity, which, however, needs to be confirmed through clinical trials including pharmacokinetic assessments.

JAKi has been most commonly associated with infectious adverse events, cardiovascular events, including thromboembolism, neoplasms, and intestinal and gastric perforation (31). Adverse events during JAKi treatment in the present study were documented for three out of five patients. Those included mild infections and, in particular, mild herpes simplex virus 1 (HSV-1) reactivations and mild respiratory tract infections. Stroke in one patients (patient 2) led to the suspension of the JAKi treatment. However, the diagnosis of APS in this patient suggested the autoimmune etiology of stroke, which would be consistent with a partial effectiveness of baricitinib in controlling STAT3-GOF-associated immune dysregulation, so that we will consider the reintroduction of a JAKi. Considering all other reviewed cases, a lethal outcome has been reported for four patients. However, for all those cases, lethal outcomes appear not to be related with JAKi treatment. Severe infections were reported in two of the cases with lethal outcome. Yet, in both those cases, severe infections might rather relate to concomitant immunosuppressive medications. Given the disease intrinsic predisposition to diverse forms of immune dysregulation, STAT3-GOF patients may necessitate long-term or even lifelong treatment with a JAKi. Prolonged treatment with JAKi, especially in adult patients with comorbidities, may be associated with risk for adverse events, especially cardiovascular events. It needs to be elucidated if those risks are relevant for patients with STAT3-GOF. We propose monitoring for cardiovascular risk and its management in all STAT3-GOF patients, even untreated ones, as this may facilitate benefit–risk assessment for the introduction or prolonged continuation of JAKi treatment.

The heterogeneity of STAT3-GOF-related disease including varying age at onset, onset patterns and severity, preexisting end-organ damage, delayed genetic diagnosis, previous or concomitant treatment with steroids, and/or immunomodulatory medications may have affected the reported efficacy of JAKi. Furthermore, concomitant immunosuppressive treatment and comorbidities, such as diabetes and preexisting lung disease, together with patient vaccination status, concomitant prophylactic anti-infective treatment, and the duration of JAKi treatment can also affect the risk for infectious and non-infectious complications. All aforementioned factors were not uniformly documented in identified published cases and may have influenced reported patient outcomes, which needs to be evaluated through studies including larger patient numbers and longer follow-up documentation.

In summary, STAT3-GOF is an IEI characterized by a broad phenotypic spectrum, including life-threatening immune dysregulation. The latter can be refractory to conventional immunomodulatory treatments, and genetic diagnosis of STAT3-GOF is a prerequisite for timely consideration of a targeted treatment. Our experience, in line with previous reports, suggests that JAKi can be an effective and safe approach for treating diverse STAT3-GOF-associated manifestations. However, clinical trials are needed to validate both the effectiveness and safety of JAKi in STAT3-GOF.

## Data Availability

The original contributions presented in the study are included in the article/**Supplementary Material**. Further inquiries can be directed to the corresponding author.

## Supplementary material

Supplementary Figure 1 Case timelines for seven patients with STAT3-GOF.

Supplementary Table 1 Studied patients’ *STAT3* variants and previous reports, including patient identification number.

Supplementary Table 2 Summary of a total of 41 patients with STAT3-GOF reported to have received JAKi treatment.

Supplementary Table 3 Summary of dose, adverse events, and efficacy of JAKi treatment in 41 patients with STAT3-GOF.
