# Supplementary material for: JAK inhibitors to treat STAT3 gain-of-function: a single-center report and literature review
Source: Front Immunol. 2024 Aug 23;15:1400348. doi: 10.3389/fimmu.2024.1400348 (PMC11377292; doi:10.3389/fimmu.2024.1400348)

Supplement

Suppl. Table 1: Patients mutations and previous reports, including patient identification number

Patient I.D.	Mutation		Previously reported by	Study I.D.	Study reference
**1**	c.[859T>G];[859=]	p.[(Leu287Val)];[(Leu287=)]	Sogkas G, et al., Int Arch Allergy Immunol. 2022;183:337-349	p. 282	9
			Leiding JW, et al., J Allergy Clin Immunol. 2023;151:1081-1095.	p. 50	2
**2**	c.[.2144C>T];[2144=]	p.[(Thr716Met)];[(Thr716=)]	Sogkas G, et al., Int Arch Allergy Immunol. 2022;183:337-349	p. 227	9
			Leiding JW, et al., J Allergy Clin Immunol. 2023;151:1081-1095.	p. 163	2
**3**	c.[.2144C>T];[2144=]	p.[(Thr716Met)];[(Thr716=)]	Sogkas G, et al., Ann Rheum Dis. 2021;80:392-399.	p. 59	13
			Leiding JW, et al., J Allergy Clin Immunol. 2023;151:1081-1095.	p. 162	2
**4**	c.[653T>C];[653=]	p.[(Val218Ala)];[(Val218=)]	Sogkas G, et al., Int Arch Allergy Immunol. 2022;183:337-349	p. 154	9
			Leiding JW, et al., J Allergy Clin Immunol. 2023;151:1081-1095.	p. 34	2
**5**	c. [1244A>G]; [1244=]	p. [(Glu415Gly)];[(Glu415=)]	n.a.	n.a.	n.a.
**6**	c.[863A>C];[863=]	p.[(Gln288Pro)];[(Gln288=)]	Leiding JW, et al., J Allergy Clin Immunol. 2023;151:1081-1095.	p. 51	2
**7**	c.[1276T>C]; [1276=]	p.[(Cys426Arg)];[(Gln426=)]	Sogkas G, et al., Int Arch Allergy Immunol. 2022;183:337-349	p. 278	9
			Leiding JW, et al., J Allergy Clin Immunol. 2023;151:1081-1095.	p. 108	2

i.d., identification number; n.a., not applicable

Suppl. Table 2

patient I.D.	STAT3 variant	localization of variant at STAT3	age at reporting	sex	reported follow-up duration	tofacitinib	ruxolitinib	baricitinib	JAKi at reporting	concurrent TCZ	previous TCZ	subsequent TCZ	HSCT	lymphadenopathy	splenomegaly	cytopenia	enteropathy	arthritis/enthesiopathy	eczema	ILD	T1D	AIH	other immune dysregulation	vital status at reporting	reference
1	p.E16A	NTD	6 m	F	n.a.	blue			blue						grey	yellow	yellow				yellow	grey	grey	alive	17
2	p. R70H	NTD	18 y	F	3 m		blue		blue					yellow		green								alive	8
3	p. R152W	CCD	8 y	M	3 m		blue		blue	blue				yellow	yellow	grey	yellow							alive	7
4	p. R152W	CCD	32 y	M	22 m	blue			blue						yellow		yellow							alive	8
5	p. K163E	CCD	8 y	M	26 m	blue			blue					red			green			yellow				alive	8
6	p. E166N	CCD	2 y	M	19 m		blue		blue						grey		green		yellow					alive	8
7	p. F174S	CCD	14 y	F	1 m		blue		blue		blue				red	red	red					red	grey	dead	7
8	p. V218A	CCD	30 y	M	10 m			blue	blue						green					green			yellow	alive	p.r.
9	p.E286G	CCD	3 y	M	8 m		blue			blue			blue				yellow						grey	dead	7
10	p. L287V	CCD	19 y	F	60 m		blue	blue	blue		blue			yellow	yellow	yellow	yellow		green				yellow	alive	p.r.
11	p.M329K	DBD	20 y	n.a.	19 m	blue			blue											yellow				alive	19
12	p.Q344H	DBD	10 y	F	42 m		blue		blue		blue				grey	green	green			yellow		green		alive	7,19
13	p. K348E	DBD	3y	M	n.a.	blue			blue					grey			grey		yellow					alive	16
14	p. Q361R	DBD	23 y	F	8 m		blue							grey	yellow	red								alive	2,8
15	p. T389R	DBD	14 y	F	42 m	blue			blue					green	yellow	green								alive	8
16	p. K392R	DBD	10 y	M	15 m		blue		blue					green	green	green			green					alive	8
17	p.V393A	DBD	22 y	F	18 m	blue			blue		blue						yellow	yellow		yellow				alive	19,21
18	p. N401D	DBD	25 y	F	12 m	blue			blue							grey	green						grey	alive	20
19	p. F408L	DBD	10 y	M	16 m	blue			blue					red		green	red			red			green	alive	8
20	p. F408L	DBD	6 y	M	4 m	blue			blue					green			green			green				alive	8
21	p. E415G	DBD	2y	M	15 m*		blue		blue	blue							yellow		yellow	yellow				alive	p.r.
22	p. E415G	DBD	4 y	F	n.a.		blue		blue								yellow			yellow	grey	yellow	grey	dead	16
23	p. E415K	DBD	11 y	F	52 m		blue		blue					green	green		green	grey	grey	green			grey	alive	8,19,22
24	p. G419R	DBD	16 y	F	32 m		blue		blue							green				green				alive	8
25	p. N420K	DBD	16 y	F	7 m		blue		blue									grey	grey				yellow	alive	2,8,23
26	p. G421R	DBD	22 y	F	11 m		blue		blue						red	grey	grey						green	alive	8,24
27	p.G421R	DBD	13 y	M	6 m	blue			blue		blue				grey	grey		yellow				grey	yellow	alive	7
28	p. G421R	DBD	3 y	M	20 m		blue		blue														green	alive	8
29	p. G618A	SH2D	10 y	F	n.a.	blue			blue								grey	yellow				yellow		alive	16
30	p. N646K	SH2D	15 y	M	36 m		blue		blue					grey	yellow				yellow	green			green	alive	2,8
31	p. N646K	SH2D	12 y	M	7 m		blue						blue					grey	grey	yellow	yellow		red	alive	8,25
32	p. K658N	SH2D	3 y	M	28 m		blue		blue							green	green			yellow	red			alive	8,26
33	p.P715L	TAD	14 y	F	n.a.	blue			blue					grey		grey		green						alive	16
34	p.P715L	TAD	10 y	F	n.a.		blue		blue							red								alive	18
35	p.P715L	TAD	15 y	M	n.a.		blue		blue			blue					red		grey	red			grey	dead	7
36	p. P715L	TAD	4 y	M	34 m		blue		blue					green					green		yellow		yellow	alive	8
37	p. P715L	TAD	5 y	M	20 m		blue		blue					green		green							green	alive	8
38	p. P715L	TAD	1 y	M	20 m		blue						blue	yellow	yellow			grey	grey				yellow	alive	2,8
39	p. T716M	TAD	14 y	F	17 m		blue		blue					green	green	green								alive	8
40	p. T716M	TAD	39 y	F	47 m			blue								yellow			green	yellow			yellow	alive	p.r.
41	p. T716M	TAD	74 y	M	60 m			blue	blue									green		green				alive	p.r.

AIH, autoimmune hepatitis; CCD, coiled-coil domain; DBD, DNA-binding domain; F, female; I.D., identification number; ILD, interstitial lung disease; m, months; M, male; n.a., not available; p.r., present report; y, years; NTD, NH2-terminal domain; SH2D, Src-homology 2 domain; TAD, transcription activation domain; T1D, type 1 diabetesTCZ, tocilizumab. Red color indicates no response, yellow color indicates partial response, green color indicates complete response, grey color indicates lack of follow-up data regarding respective manifestation of STAT3-GOF.

*18 months , including 3 months of paused treatment

Suppl. Table 3

Patient I.D.	**STAT3 variant**	**tofacitinib**	**Ruxolitinib**	**Baricitinib**	Daily dose	Divided doses per day	Reported adverse events and outcome	Comment on efficacy of JAKi
1	**p.E16A**	■			n.a.	n.a.	not reported	"sustained improvement in her glucose levels, pancytopenia, and enteropathy symptoms"
2	**p. R70H**		■		8 $mg/m^2/day$	1	not reported	cytopenia: complete response, lymphadenopathy: partial response
3	**p. R152W**		■		30 $mg/m^2/day$	2	transient mild rise in ALT, outcome: resolved 3 weeks after starting JAKi	"decrease in hepatosplenomegaly, weight gain, remomed NG tube"
4	**p. R152W**	■			10 mg/day	2	not reported	splenomegaly and enteropathy: partial response
5	**p. K163E**	■			10 mg/day	2	not reported	lymphadenopathy: no response, enteropathy: complete response
6	**p. E166N**		■		51 $mg/m^2/day$	2	Varicella, no dose reduction/pause, outcome: resolved	enteropathy: complete response, eczema: partial response
7	**p. F174S**		■		10 $mg/m^2/day$	2	continued DIC and multiorgan failure, patient reported dead	"improvement: none"
8	**p. V218A**			■	4 mg/day	1	none	splenomegaly and ILD: complete response, trachyonychia and alopecia: partial response
9	**p.E286G**		■		30 $mg/m^2/day$	2	influenza, *E.coli* gastroenteritis, patient reported dead (cause of death related to HSCT)	"TPN discontinued within 2 months, no HLH flares", however treated with HSCT, suggesting partial response to JAKi
10	**p. L287V**		■	■	4 mg/day*/ 28 $mg/m^2/day$**	1 / 2	herpes labialis, recurrent bronchitis and sinusitis, mild gastrointestinal symptoms, outcome: infections resolved after stopping TCZ	splenomegaly, lymphadenopathy, cytopenias and enteropathy: partial response, eczema: complete response
11	**p.M329K**	■			20 mg/day	2	not reported	"stable clubbing and retractions, hypoxia and dyspnea resolved", "ground-glass opacities improved", "inflammatory arthritis, which also improved with JAK inhibition"
12	**p.Q344H**		■		30 $mg/m^2/day$	2	mild elevation in bilirubin, psoriasis (on dual TCZ and JAKi treatment)	"TPN discontinued within 3 weeks, transaminitis resolved, off continuous oxygen support within one month, resolved cytopenias"
13	**p. K348E**	■			7.5 mg/day	1	not reported	"skin rash improved instantaneously"
14	**p. Q361R**		■		20 $mg/m^2/day$	2	anemia (preexisting), neutropenia (without	treatment refractory cytopenias leading to suspension of JAKi

							LGL relapse), JAKi stopped	
15	**p. T389R**				10 mg/day	2	not reported	lymphadenopathy and cytopenia: complete response, splenomegaly: partial response
16	**p. K392R**				17 mg/m²/day	2	not reported	lymphadenopathy, splenomegaly, cytopenias and eczema: complete response
17	**p.V393A**				5 mg/day	1	not reported	improvement of lung function and stable radiological findings parameters after JAKi, “enteropathy and inflammatory arthritis, which also improved with JAK inhibition”
18	**p. N401D**				15 mg/day	2	not reported	"budesonide discontinued after 2 months"
19	**p. F408L**				15 mg/day	3	unintentional weight gain, JAKi dose reduction	lymphadenopathy, enteropathy and ILD: no response, cytopenias: complete response
20	**p. F408L**				10 mg/day	2	not reported	lymphadenopathy, enteropathy and ILD: complete response
21	**p. E415G**				16 mg/m²/day	3	none	enteropathy, eczema and ILD: partial response
22	**p. E415G**				5 mg/day	n.a.	not reported, however, patient reported dead	"RUXO contributed tot he alleviation of enteropathy, IP, and chronic liver disease", “she died at the age of 8 years, which was unrelated to the STAT3-GOF symptoms”
23	**p. E415K**				20 mg/m²/day	2	thoracic herpes zoster	lymphadenopathy, splenomegaly, enteropathy and ILD: complete response
24	**p. G419R**				40 mg/m²/day	2	headache, vomiting; dose reduction	cytopenia and ILD: complete response
25	**p. N420K**				30 mg/m²/day	2	not reported	mouth lesions and tongue ulcerations: partial response
26	**p. G421R**				20 mg/m²/day	2	not reported	splenomegaly: no response, "other autoimmunity of dysregulation": complere response
27	**p.G421R**				5 mg/day	1	vomiting, dose decreased to 5 mg/day	"no worsening of arthritis and scleroderma"
28	**p. G421R**				8 mg/m²/day	2	not reported	"other autoimmunity of dysregulation": complete response
29	**p. G618A**				10 mg/day	n.a.	not reported	"after tofacitinib initiation, her arthralgia and liver dysfunction improved and the dose of prednisolone was reduced"
30	**p. N646K**				59 mg/m²/day	2	unintentional weight gain, no JAKi change	ILD: complete response, splenomegaly and eczema: partial response
31	**p. N646K**				30 mg/m²/day	2	not reported	ILD and T1D: partial response, "other autoimmunity of dysregulation": no response
32	**p. K658N**				38 mg/m²/day	3	bacterial sepsis, temporal epilepsy	"chronic diarrhea resolved, allowing rapid catch-up growth"

33	**p.P715L**				10 mg/day	n.a.	not reported	"After tofacitinib initiation, her arthralgia improved, and through magnetic resonance imaging, it could be seen that the bone marrow edema of the fingers disappeared"
34	**p.P715L**				n.a.	n.a.	severe thrombocytopenia	"JAK inhibitor was discontinued after the appearance of severe thrombocytopenia (managed with intravenous immunoglobulins and high-dose corticosteroids)"
35	**p.P715L**				15 mg/m²/day	2	raised ALT and gGT, patient reported dead, cause of death: respiratory failure complicated by pulmonary hemorrhage with aspergillosis, torulopsis glabatra and pseudomonas sepsis	"improved feeding tolerance", "worsened respiratory status and enteropathy" led to TCZ after ruxolitinib
36	**p. P715L**				40 mg/m²/day	2	not reported	"maintained resolution of panniculitis and lymph node swelling", "with the beginning of ruxolitinib treatment, the fatty tissue returned well in areas where insulin had not been injected before", lymphadenopathy and eczema: complete response, T1D and "other autoimmunity of dysregulation": partial response
37	**p. P715L**				10 mg/m²/day	2	unintentional weight gain, no JAKi change	lymphadenopathy, cytopenia, "other autoimmunity of dysregulation": complete response
38	**p. P715L**				54 mg/m²/day	2	mild viral infections (astrovirus gastroenteritis, adenovirus and human metapneumovirus respiratory infection), reported dead (cause of death related to HSCT)	lymphadenopathy, splenomegaly, "other autoimmunity of dysregulation": partial response
39	**p. T716M**				17 mg/m²/day	3	unintentional weight gain, JAKi dose reduction	lymphadenopathy, splenomegaly, cytopenia: complete response
40	**p. T716M**				4 mg/day	1	herpes labialis, mild bronchitis, unintentional weight gain, stroke (APS), JAKi was stopped due to stroke	ILD and cytopenia: partial response, eczema: complete response
41	**p. T716M**				2 mg/day	1	mild bronchitis	ILD and arthritis: complete response

APS, antiphospholipid syndrome; DIC, disseminated intravascular coagulation; F, female; HLH, hemophagocytic lymphohistiocytosis; HSCT, hematopoietic stem cell transplantation; I.D., identification number; ILD, interstitial lung disease; IP, interstitial pneumonia; LGL, large granular lymphocytic leukemia; m, month; M, male; n.a., not available; NG, nasogastric; PEG, percutaneous endoscopic gastrostomy; RUXO, ruxolitinib; T1D, type 1 diabetes; TCZ, tocilizumab; TPN, total parenteral nutrition; y, years

* baricitinib dose

** ruxolitinib dose

Suppl. Figure 1

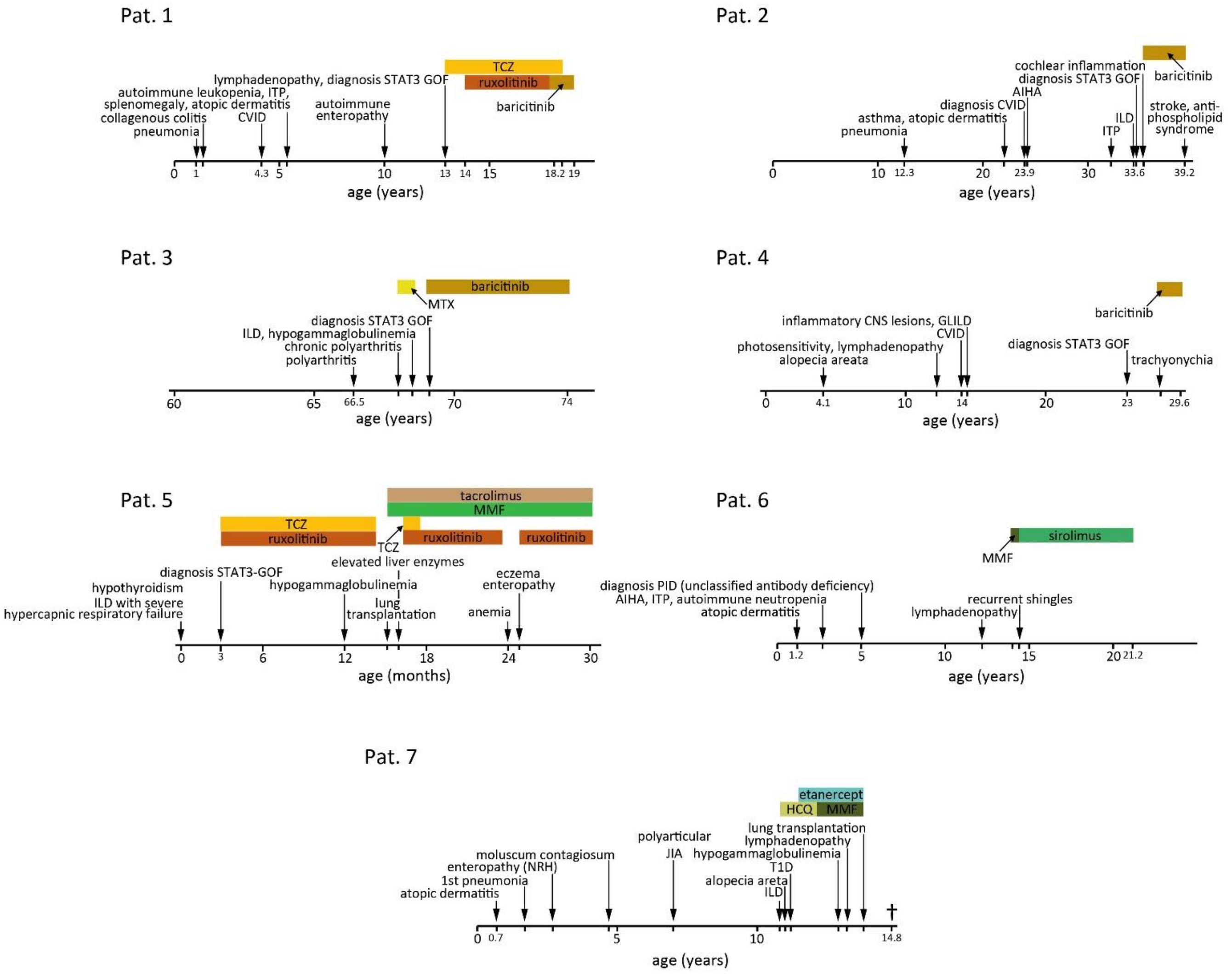

Supplement: Supplementary Figure 1 — Case timelines for seven patients with STAT3-GOF. [file DataSheet1.pdf]
